# High resolution spectral metrology leveraging topologically enhanced optical activity in fibers

**DOI:** 10.1038/s41467-020-18931-6

**Published:** 2020-10-16

**Authors:** Aaron P. Greenberg, Gautam Prabhakar, Siddharth Ramachandran

**Affiliations:** 1 Boston University, Boston, MA 02215 USA; 2 Present Address: Aeva Inc, Mountain View, CA 94043 USA

**Keywords:** Fibre optics and optical communications, Optical physics, Optical techniques, Optical metrology

## Abstract

Optical rotation, a form of optical activity, is a phenomenon employed in various metrological applications and industries including chemical, food, and pharmaceutical. In naturally-occurring, as well as structured media, the integrated effect is, however, typically small. Here, we demonstrate that, by exploiting the inherent and stable spin-orbit interaction of orbital angular momentum fiber modes, giant, scalable optical activity can be obtained, and that we can use this effect to realize a new type of wavemeter by exploiting its optical rotary dispersion. The device we construct provides for an instantaneous wavelength-measurement technique with high resolving power *R* = 3.4 × 10^6^ (i.e., resolution < 0.3 pm at 1-μm wavelengths) and can also detect spectral bandwidths of known lineshapes with high sensitivity.

## Abstract (web summary)

Optical rotation in a medium, which is typically small, can be enhanced by harnessing spin-orbit interaction in fiber modes. Here the authors use this effect to develop a fiber-based wavelength-measurement technique with high resolution.

## Introduction

**Fig. 1:**
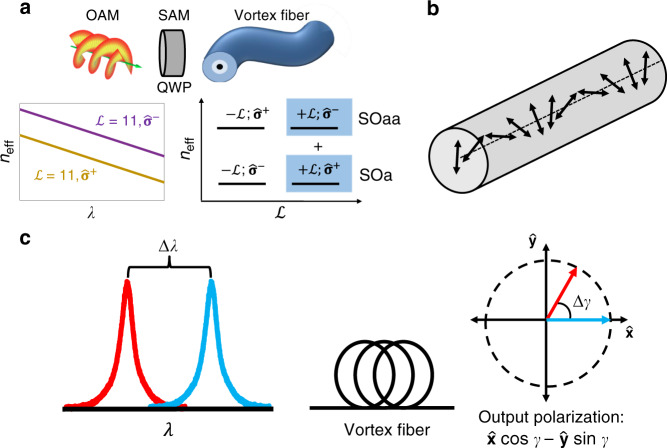
Vortex fiber superposition phenomena. **a** Spin orbit interaction (SOI) between OAM (image from E-Karimi is reproduced under a CC BY-SA license) and SAM (imparted by quarter wave plate, QWP) splits effective index (*n*_eff_) of SOa and SOaa states in a fixed-$|{\cal{L}}|$ 4-D vortex fiber mode Hilbert space. Numerical simulation reveals the circular birefringence between OAM ${\cal{L}} = + 11$ modes with opposite $\widehat {\mathbf{\sigma }}$. The superposition of the highlighted blue states is a linearly polarized OAM beam; which, when launched into the vortex fiber (reproduced from ref. ^13^, 2015, Optical Society of America), experiences optical activity. **b** Illustrative depiction of optical activity, where a linearly polarized beam rotates while propagating through the vortex fiber. **c** The wavelength dependence of optical activity, known as optical rotary dispersion, means a shift in wavelength (Δ*λ*) rotates the output linear polarization (Δ*γ*).

It has been well established that light with helical phase fronts carry orbital angular momentum (OAM) as designated with $\pm {\!}{\cal{L}}$ topological charge^1,2^, which defines the number of light’s twists per wavelength and directional handedness. In recent years OAM beams have risen to prominence for their applications in STED microscopy^3^, laser machining^4^, advanced manufacturing^5^, optical tweezers^6^, and both classical and quantum communications^7–9^. These beams can also carry spin angular momentum (SAM), as determined by their circular polarization, where left or right is denoted by $\widehat {\mathbf{\sigma }}^ \pm = \left( {\widehat {\mathbf{x}} \pm i\widehat {\mathbf{y}}} \right)/\sqrt 2$, respectively. The two quantities, ${\cal{L}}$ and $\widehat {\mathbf{\sigma }}$, and how they are paired together, define a 4-dimensional Hilbert space of given $|{\cal{L}}|$ in optical fibers (Fig. 1a), represented by the electric fields^10^:

1 $${\mathbf{E}}\left( {r,\phi ,z,t} \right) = F\left( r \right)\left\{ {\begin{array}{*{20}{c}} {\widehat {\mathbf{\sigma }}^ \pm \exp \left( { \pm i{\cal{L}}\phi } \right)\exp \left( {i\beta _{{\mathrm{SOa}}}z} \right)} \\ {\widehat {\mathbf{\sigma }}^ \pm \exp \left( { \mp i{\cal{L}}\phi } \right)\exp \left( {i\beta _{{\mathrm{SOaa}}}z} \right)} \end{array}} \right\}e^{ - i\omega t}$$

where *F*(*r*) is the mode amplitude distribution, *ϕ* is the azimuthal angle, *ω* is the angular frequency, and *β* is the propagation constant, which is proportional to the fiber mode effective index (*n*_eff_) and wavelength (*λ*) by $\beta = 2\pi \cdot n_{{\mathrm{eff}}}/\lambda$. The distinct propagation constants, *β*_SOa_ and *β*_SOaa_, represent two degenerate spin-orbit aligned states (SOa) where ${\cal{L}}$ and $\widehat {\mathbf{\sigma }}$ are of the same sign, and two degenerate spin-orbit anti-aligned states (SOaa) where these quantities are of opposite sign. The degeneracy within this Hilbert space is lifted between these SOa and SOaa pairs due to light confinement in a waveguide, and by the spin-orbit interaction (SOI) of light, which describes the interaction between OAM and SAM^11^ in an attractive potential. The spatial inhomogeneity of the ring-core refractive index profile *n*(*r*) of fibers designed for OAM mode propagation^12,13^, introduces geometrodynamic polarization-dependent perturbations that splits effective index (Δ*n*_eff_) depending on $\widehat {\mathbf{\sigma }}^ \pm$:

2 $${\Delta}n_{\mathrm{eff}} \propto {\cal{L}}{\int} {F^2} \left( r \right)\frac{{\partial {\Delta}n\left( r \right)}}{{\partial r}}dr$$

where Δ*n*(*r*) is the vortex fiber’s *n*(*r*) profile, relative to the index of its cladding. The intrinsic OAM of the modes amplifies this effect, enabling large effective index differences Δ*n*_eff_ > 10^−4^ between $\widehat {\mathbf{\sigma }}^ +$ and $\widehat {\mathbf{\sigma }}^ -$ OAM modes with the same topological charge ${\cal{L}}$. Ergo, the SOI of light is responsible for splitting the effective indices of SOa and SOaa modes, resulting in circular birefringence^14^ (Fig. 1a). A superposition state of two modes with the same ${\cal{L}}$ but opposite $\widehat {\mathbf{\sigma }}$ (i.e., a SOa and SOaa state) effectively yields a linearly polarized beam with topological charge ${\cal{L}}$. Hence, the SOI-induced circular birefringence results in optical activity (OA), which manifests as a rotation of the beam’s linear polarization orientation angle as it propagates down the fiber (see Eq. (3) and Fig. 1b). For an input with a $\widehat {\mathbf{x}}\left( { = \widehat {\mathbf{\sigma }}^ + + \widehat {\mathbf{\sigma }}^ - } \right)$ polarized OAM superposition of topological charge ${\cal{L}}$, the fiber output field would be:

3 $${{\mathbf{E}}\left( {r,\phi ,z,t} \right)} 	= {F\left( r \right)\exp \left( { - i\omega t} \right)\exp \left( {i{\cal{L}}\phi } \right)\exp \left( {i\bar \beta Ƶ} \right)\left\{ {\widehat {\mathbf{\sigma }}^ + e^{i\gamma } + \widehat {\mathbf{\sigma }}^ - e^{ - i\gamma }} \right\}} \\ 	= {\sqrt 2 F\left( r \right)\exp \left( { - i\omega t} \right)\exp \left( {i{\cal{L}}\phi } \right)\exp \left( {i\bar \beta Ƶ} \right)\left\{ {\widehat {\mathbf{x}}\cos \gamma - \widehat {\mathbf{y}}\sin \gamma } \right\}}$$

where Ƶ is the fiber (interaction) length, $\bar \beta$ is an average propagation constant $\bar \beta = \left( {\beta _{{\mathrm{SOa}}} + \beta _{{\mathrm{SOaa}}}} \right)/2$ and *γ* is the output OA polarization angle:

4 $$\gamma = Ƶ \cdot \left( {\beta _{{\mathrm{SOa}}} - \beta _{{\mathrm{SOaa}}}} \right)/2 = Ƶ \cdot \pi \cdot {\Delta}n_{{\mathrm{eff}}}/\lambda$$

The polarization angle *γ* exhibits wavelength dependence, known as optical rotary dispersion (ORD) (Fig. 1c), which has played roles in quantum metrology^15^ and in ascertaining molecular conformation^16^. Although all OA media, either naturally occurring^17,18^, or structured materials^19–22^, share these traits, they often have interaction lengths limited to between μm and cm, therefore inhibiting any of these systems from achieving scalable OA strength.

Here we show that OAM vortex fibers circumvent this scalability limitation and allow for high-resolution spectral metrology. Vortex fibers have demonstrated support of OAM modes over long interaction lengths, up to km-length propagation^23^, providing for considerable scalability not afforded by other media. We also find that the interference that produces the OA in vortex fiber is inherently stable, for reasons related to the nature of spin-orbit interactions that yield mode splittings. This preserves the OA effect over long fiber lengths even in perturbative settings. By employing this OAM superposition state to obtain giant OA, we can observe fine changes in wavelength, making it the effect that forms the basis for a high-resolution wavemeter, capable of single-shot dominant wavelength measurements, and fine spectral bandwidth detection.

## Results

### Wavemeter resolution

The inherent optical rotary dispersion of OAM superposition modes is the phenomenon used to establish a wavelength to polarization spectroscopic mapping. In order to see this, we take a first-order Taylor expansion of Eq. (4), yielding:

5 $$\gamma = Ƶ \cdot \pi \cdot {\Delta}n_{{\mathrm{eff}}}/\lambda \to {\Delta}\gamma = - \left\{ {Ƶ\pi \frac{{{\Delta}n_{\mathrm{g}}}}{{\lambda ^2}}} \right\}{\Delta}\lambda = \alpha {\Delta}\lambda$$

where Δ*n*_g_ is the difference in group index between SOa and SOaa modes, Δ*λ* is a change in wavelength, and *α* is a condensed factor defining the integrated birefringent strength of our ORD. Evidently, any change in wavelength linearly maps to a change in polarization angle. Using OAM fiber modes, we can substantially increase the magnitude of *α*, hence enhancing resolving power.

**Fig. 2:**
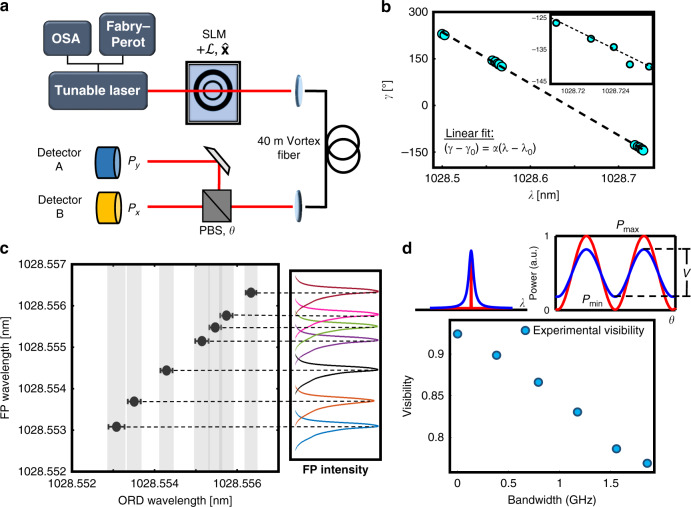
ORD wavemeter performance. **a** A SLM generated ${\widehat {\mathbf{x}}}$-polarized OAM superposition state experiences OA through 40 m of vortex fiber. At the output, a PBS splits power into two orthogonal polarization bins (${\widehat {\mathbf{x}}}$, ${\widehat {\mathbf{y}}}$), and the ratio of powers, (*P*_x_, *P*_y_) instantaneously determines polarization orientation angle *γ* (see Methods). **b** Calibration of ORD birefringent strength (*α*) is necessary to accurately map *λ* to *γ*. Wavelength sweep Δ*λ*, resolved using a Fabry–Perot (FP) reference, has a corresponding polarization rotation Δ*γ*, measured by power detectors. Reference values *λ*_0_, measured by an optical spectrum analyzer (OSA), and *γ*_0_ are added to convert measurements to absolute values. Data are collected over three spectral regimes (Inset focuses on one regime) and *α* is taken as the slope of an applied linear fit. **c** Resolution of the ORD wavemeter is determined by changing wavelength with a TEC in sub-picometer steps. The gray bands indicate detector dependent measurement error (s.d.). Using OAM ${\cal{L}}$ = +11 and 40-m vortex fiber we find the ORD wavemeter can measure down to Δ*λ* < 0.3 pm at 1028 nm (*R* = 3.4 × 10^6^) with nearly full confidence thresholding. **d** ORD wavemeter visibility (*V*) reduces with broadening of laser linewidth. Red represents the visibility of a near-monochromatic laser, and blue represents a broadened spectrum. Measured visibility as a function of bandwidth reveals a monotonically decreasing relationship as the source is broadened by ~2 GHz.

We use a tunable CW laser at 1028 nm with 100-kHz linewidth, which is transformed to the ${\cal{L}}$ = +11; $\widehat {\mathbf{\sigma }}^ + + \widehat {\mathbf{\sigma }}^ -$ (or $\widehat {\mathbf{x}}$-polarized) OAM superposition state with a spatial light modulator (SLM) and coupled to a 40-m vortex fiber^13^ (Fig. 2a). Output mode spatial interferometry analysis^24^ indicates >20-dB mode purity and >16-dB polarization extinction. A polarization beam splitter (PBS) projects the output beam into two orthogonal polarization bins ($\widehat {\mathbf{x}}$, $\widehat {\mathbf{y}}$) and corresponding powers (*P*_x_, *P*_y_) are measured with photodetectors. The ratio of $P_{\mathrm{x}} \propto \cos ^2\left( \gamma \right)$ and $P_{\mathrm{y}} \propto \sin ^2\left( \gamma \right)$ (see Eq. (3)) yields *γ*:

6 $$P_{\mathrm{y}}/P_{\mathrm{x}} = \tan ^2\left( \gamma \right) \to \gamma = \tan ^{ - 1}\left( {\sqrt {P_{\mathrm{y}}/P_{\mathrm{x}}} } \right)$$

The simplicity of Eq. (6) means *γ* can be read-out instantaneously, requiring no post-processing, and allows a unique mapping of wavelength to polarization angle between an angular free-spectral-range (FSR) of 0 and 90°. For the ${\cal{L}} = 11$ OA state, this is equivalent to a wavelength FSR of Δ*λ* = 46 pm. To calibrate the device, we plot polarization rotation ${\Delta}\gamma = \left( {\gamma - \gamma _0} \right)$ versus a corresponding change in wavelength ${\Delta}\lambda = \left( {\lambda - \lambda _0} \right)$, where *γ*_0_ and *λ*_0_ are two initial reference states (see Eq. (5) and Fig. 2b). The slope of a linear fit applied to this plot is our calibrated *α* factor, completing our *λ* to *γ* mapping (see Methods). To determine the resolving power of the ORD wavemeter, we finely tune our laser wavelength with a thermoelectric cooler (TEC) and measure *γ*. Using our calibrated *α* factor, we can convert the changes in polarization Δ*γ* to changes in wavelength Δ*λ*, which we validate using a FP reference that simultaneously measures wavelength. The non-overlapping nature of the gray error bands in Fig. 2c confirm wavelength shifts down to at least 0.3 pm are readily discernable by our device. Since the measurement only involves recording power, the speed, noise performance, robustness, and reliability of the ORD wavemeter depends largely on the choice of photodetectors used (see Methods).

### Spectral bandwidth detection

So far, we have shown the capability to measure wavelength to sub-picometer resolution, but the ORD wavemeter can detect spectral bandwidth as well^25^. By biasing the PBS angle *θ* in our setup to maximize and minimize powers in the two detectors (*P*_max_, *P*_min_), the bandwidth of the source may be measured from the visibility ($V = P_{{\mathrm{max}}} - P_{{\mathrm{min}}}/P_{{\mathrm{max}}} + P_{{\mathrm{min}}}$) of the ORD wavemeter. Linewidth broadening introduces additional spectral components that experience OA to differing degrees. The superposition of all these OA states turns the output into an elliptically polarized beam that reduces interferometric visibility (Fig. 2d). With a 50-m vortex fiber we experimentally observe a clear monotonically decreasing relationship between bandwidth and visibility (Fig. 2d). Considering we should be able to measure visibility down to *V* ≈ 0, the small visibility change suggests total broadening detection much larger than the 2 GHz (~7 pm) we could measure with existing infrastructure. ORD wavemeter bandwidth detection could also be extended to measure larger spectral broadening by shortening fiber length and/or using lower order modes. This would change the power distribution of (*P*_max_, *P*_min_) and allow the ORD wavemeter to detect larger changes in bandwidth for the similar drops in visibility. In general, knowing the input functional form of the broadened source allows the measured visibility of the ORD wavemeter to be uniquely mapped to spectral bandwidth (simulations discussed in Supplementary Note 4).

### Environmental stability

**Fig. 3:**
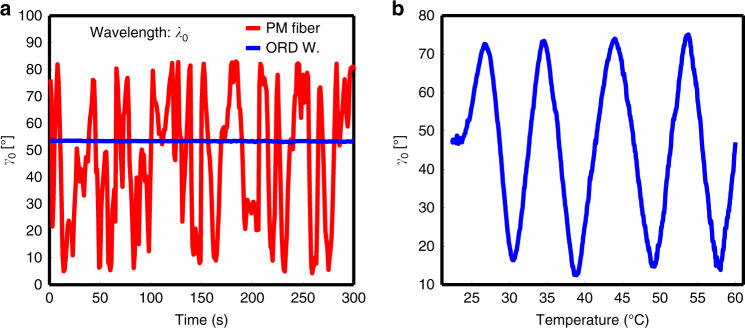
Stability metrics. **a** Stability of the ORD wavemeter at a single wavelength (*λ*_0_) in a mechanically perturbed environment, where the vortex fiber is shaken over a 5 min period. The change in OA angle, *γ*_0_, due to these perturbations is barely resolvable by our Fabry–Perot (< 0.4 pm). This is in contrast to the apparently unstable behavior of a birefringent PM-fiber wavemeter. **b** Stability of the ORD wavemeter at a single wavelength (*λ*_0_) in a thermally perturbed environment, where the vortex fiber is heated from 22 °C to 60 °C. The thermal expansion of the fiber causes the OA angle to change with very systematic behavior. The mechanical and temperature stability tests both demonstrate how inherently resilient the ORD wavemeter is to perturbed environments, unlike the PM-fiber which showed erratic instability.

We test the stability of the ORD wavemeter with respect to mechanical vibrations and temperature fluctuations. Figure 3a reveals that the measured reference rotation angle *γ*_0_ (blue trace) remains invariant with time, even when subjected to mechanical vibrations. This is especially evident when compared with a similar experiment conducted with a conventional polarization–maintaining (PM) birefringent fiber of similar length, which can also be used to map polarization state to wavelength^26^, but which displays complete instability (red trace). We also varied the temperature experienced by our device from 22 °C to 60 °C, and observe an oscillatory change in *γ*_0_ (Fig. 3b). This is primarily due to fiber expansion, which changes Ƶ in Eq. (4), leading to periodic change in *γ*_0_ (Eq. (6)). Hence, being predicable and systematic, ORD measurements with vortex fibers behaves dramatically differently from the chaotic behavior displayed by the PM fiber or, as we will discuss, other known interference-based systems.

## Discussion

The high resolving power of the demonstrated ORD wavemeter arises from the ability to obtain scalable OA in long lengths of vortex fibers, which, by definition, requires the stable propagation of superposition states. Singlet OAM states, as described by Eq. (1), are known to be stably propagated in vortex fibers^7,13^. But here, OA, provided by the $\widehat {\mathbf{\sigma }}^ \pm$ superposition of singlet OAM states, is essentially an interference phenomenon, which would usually be sensitive to environmental perturbations. For instance, even in cm-long conventional multimode fibers, modal coupling and speckle effects are well known. In fact, instability and loss of preservation of superposition states is a common debilitating feature of multiple systems (qubit-environment interactions^27^, communications in turbulent atmospheres^28^, speckle pattern spectrometers^29^, etc.). So the key question then arises – why is a superposition state in the vortex fiber stable under perturbed conditions, in contrast with expectations of other known interference phenomena? We surmise that the reason lies in the topological nature of vortex fiber birefringence. Whereas other means of yielding OA in potentially length-scalable fibers rely on material/strain effects or structured fabrication, the SOI-induced Δ*n*_eff_ splitting is inherently resistant to non-topological perturbations such as temperature, vibrations, and bends. This is to say, fiber perturbations would indeed affect the phase of the OAM eigenmodes^30^, but such a phase change is identical for both SOa and SOaa modes that form the superposition yielding OA. Support for this reasoning is available by studying the form of SOI splitting (Eq. (2)). Topologically enhanced OA depends on the spatial overlap of the field (identical for SOa and SOaa modes) and the fiber’s index profile gradient, $\partial {\Delta}n\left( r \right)/\partial r$, rather than the index profile Δ*n*(*r*) itself. The expected change that non-thermal perturbations have on the index gradient should be a second or higher order effect, and hence smaller. The experiments indicate that they are, in fact, negligible. The inherent stability of OA in the vortex fiber is key in obtaining scalability. In fact, this demonstration was limited to 40 m of fiber because our commercial FP interferometer could not provide higher resolution reference for comparison. Since OAM mode propagation up to 13.4 km^23^ has been shown to be feasible in vortex fibers, we speculate that revolving powers *R* two orders-of-magnitude higher than that demonstrated here are accessible by this device schematic.

**Fig. 4:**
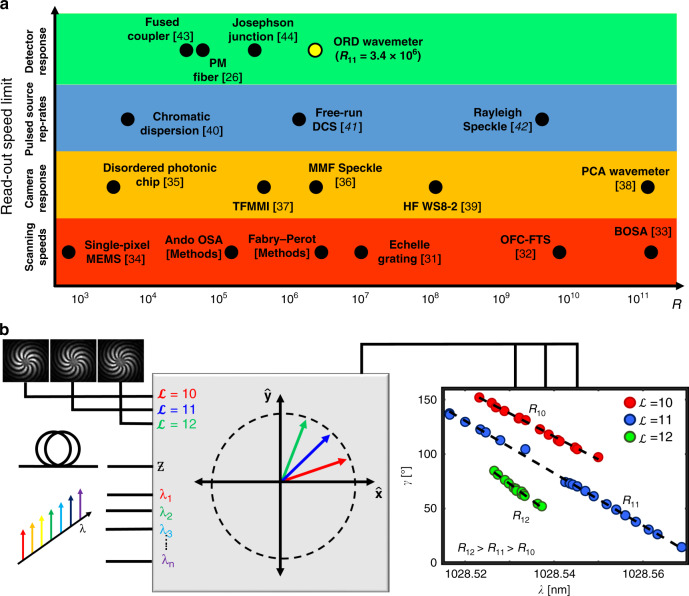
Spectroscopic application space and multiplexing. **a** Performance metric chart of various spectroscopic devices – wavemeters, spectrometers, interferometers – using resolving power and categorized speed constraints. Red (bottom) band is for scanning devices, generally with mechanically moving parts. Orange is for devices that measure spatial intensity distributions with cameras. Devices whose speed depend on the pulse repetition rate of its laser source(s) are in the blue band. Lastly, green (top band) is for single-shot devices, which are principally speed constrained by the response time of their high-speed photodetectors. The ORD wavemeter (indicated in gold) demonstrates the highest resolving power of a device belonging in the green band. Abbreviations listed in Supplementary Note 7. **b** Multiplexing capabilities of the ORD wavemeter. Required inputs are OAM mode order ${\cal{L}}$, vortex fiber length, and a wavelength sweep. For OAM of different ${\cal{L}}$ there are different strengths of optical rotation with wavelength, hence distinct *α* and resolutions (*R*_10_, *R*_11_, and *R*_12_) can be identified. The multitude of available OAM modes in vortex fibers could be employed to develop generalized, multiplexed spectral measurement devices across wider wavelength ranges.

It is instructive to compare the performance of the ORD wavemeter with existing alternative approaches. We do this in Fig. 4a, which shows resolving power of select representative spectroscopic devices distinguished in 4 distinct bands based on underlying technology. The first (red) band contains techniques generally comprising scanning/moving parts such as gratings^31^, cavity mirrors^32^, probes^33^, and micro-electro mechanical systems^34^ (MEMS). The second (orange) band contains devices that map wavelength to spatial position, then measured with a camera^35–39^. Devices that exploit the temporal response of a pulse^40–42^, but whose speed would depend on specific pulse widths and shapes used, are shown in the third (blue) band. Finally, devices shown in the fourth (green) band perform the entire measurement using one, or a few, (single-pixel) detectors^43,44^ – this category includes the use of PM fibers^26^ (*R* ~ 10^4^), as well as our ORD wavemeter (*R* > 10^6^, that leverages fiber length scalability due to SOI). Devices in this green band are very promising for a range of high-speed monitoring and metrological applications, since single photodetectors can have bandwidths up to 100 GHz in the telecommunications window, yielding, potentially, ~ps response times.

An additional benefit of the ORD wavemeter is that the vortex fiber can support multiple OAM modes of differing ${\cal{L}}$ simultaneously, making it ideal for multiplexing functionalities. In this vortex fiber, Δ*n*_eff_ scales as ${\cal{L}}^2$ (see Eq. (2)), due to the combined effect of SOI (factor of ${\cal{L}}$) and the interaction of the optical field with the index inhomogeneity of the fiber$\left( {{\int} {F^2} \left( r \right)\frac{{\partial {\Delta}n\left( r \right)}}{{\partial r}}dr \propto {\cal{L}}} \right)$^45^. As such, ORD birefringent strength, *α*, scales with mode order, as demonstrated in Fig. 4b, which shows distinct resolving powers (*R*_10_, *R*_11_, and *R*_12_) for the different OAM topological charges. With multiple detectors and well-known OAM mode sorting techniques^46,47^ the schematic remains single-shot in nature. This is akin to deducing multiple unknowns given multiple measurands, as often deployed in multiparameter sensing. Given that vortex fibers supporting as many as 24 modes are now available^48^, such multiplexing schematics could lead to additional functionalities, such as removing the FSR-to-resolution tradeoffs typical of interferometric devices, while remaining single-shot and high resolution.

## Methods

### ORD wavemeter construction and experimental setup

Supplementary Fig. 1 illustrates a fully detailed ORD wavemeter setup. Our experiments used a tunable, fiber-coupled, external cavity laser (ECL, Toptica DL-pro) with narrowband < 100 kHz linewidth. The laser is passed through an optical isolator, preventing back reflections, and a polarization controller (Polcon), allowing arbitrary control of polarization. A 3-dB coupler redirects the ECL to multiple paths, two of which (back-reflection and forward) couple to an optical spectrum analyzer (OSA, Ando AQ6317B) and a Fabry–Perot scanning interferometer (FP, Thorlabs SA210-8B) (further specifications in next section). The other forward path projects the laser to free-space, which we convert to an OAM beam with topological charge ${\cal{L}}$ using a fork-pattern hologram generated on a spatial light modulator (SLM, Hamamatsu x10468-08)^13^. Since the SLM is $\widehat {\mathbf{x}}$-polarization sensitive, we maximized the power of $\widehat {\mathbf{x}}$-polarized light using a combination of the Polcon and a polarization beam splitter (PBS). When our input $\widehat {\mathbf{x}}$-polarized OAM beam couples to our optically active vortex fiber, propagation rotates the linear polarization to a new orientation (see Eq. (3)), as determined by the fiber length and OAM mode order. For our resolution experiments, we used a 40-m fiber and mode order of ${\cal{L}} =$ +11. At the fiber output, a beam splitter (BS) sends part of the beam to a Thorlabs DCC1545M camera for modal purity analysis^24^. An output PBS divides the beam into two orthogonal polarization bins aligned with ($\widehat {\mathbf{x}}$, $\widehat {\mathbf{y}}$). In the $\widehat {\mathbf{x}}$-bin, we send the beam to an output SLM and iris aperture to perform mode conversion (see Supplementary Note 3 for details). The $\widehat {\mathbf{y}}$-bin also undergoes mode conversion, but with the addition of a half wave plate (HWP) to change polarization from $\widehat {\mathbf{y}}$ to  $\widehat {\mathbf{x}}$. Following mode conversion, two low noise Ge photodetectors (Agilent HP 81521B) measure each polarization bin’s respective powers *P*_x_ and *P*_y_, which are used to calculate the OA polarization angle *γ* (see Eq. (6)) for our ORD wavemeter measurements. Slight setup variations are used for spectral broadening and stability experiments, as discussed in Supplementary Notes 4 and 5.

### Calibration method, accuracy, and noise characterization

To calibrate our device and complete our spectroscopic mapping, we simply need to determine our ORD birefringent strength, *α*, such that the linear relationship ${\Delta}\gamma = \alpha {\Delta}\lambda$ is satisfied (see Eq. (5)). For our calibration, we measure rotations in polarization ${\Delta}\gamma = (\gamma - \gamma _0)$ corresponding to changes in wavelength ${\Delta}\lambda = (\lambda - \lambda _0)$ over three mode-hop free spectral regimes of the ECL [see Fig. 2b]. The reference, or initial, wavelength, *λ*_0_ was measured using a commercial high-resolution OSA, which provided NIST-traceable absolute wavelength measurements with 10-pm resolution. Accordingly, the reference angle *γ*_0_ is the initial polarization state of the output OA beam at *λ*_0_. Subsequent relative wavelength measurements *λ* were obtained with a commercial FP with ~ 0.25-pm resolution, and polarization angles *γ* were measured from Eq. (6) with photodetectors. Since Eq. (5) limits the angular FSR of the ORD wavemeter (using a single mode) to a polarization mapping between 0 to 90° (equivalent to a wavelength FSR Δ*λ* = 46 m with the ${\cal{L}} = 11$ OA mode), the *γ* measurements in each regime are unwrapped (with further wavelength validation from both the Fabry–Perot and OSA) past these limits, such that they form the linear relation as described in Eq. (5). The calibration factor *α* is simply the slope of a linear fit applied to the measurements described, and once established, can be used to accurately convert *γ* to *λ*. When applying the linear fit, outliers arising from in-fiber mode coupling and detector noise floors were not included. Since the ORD wavemeter demonstrates considerable stability, as demonstrated in Fig. 3 and discussed in Supplementary Note 5, once the device is calibrated we can expect that *α* will remain unchanged and reliable, even in perturbed environments.

After calibration, we require another test to determine the accuracy of the ORD spectroscopic mapping. At two mode-hop free spectral regimes, we measure wavelength deduced from the ORD wavemeter using our calibration factor and plot versus the presumed ground truth as measured by the FP (see Supplementary Fig. 2). Root-mean-squared error analysis around the ground truth slope = 1 line reveals that our calibrated device is capable of predicting wavelength with an accuracy of ~1 pm. As such, the ORD wavemeter is capable of high accuracy in determining wavelength after being appropriately calibrated.

For the Agilent HP 81521B Ge detectors used in this work, the estimated noise equivalent power was <50 pW (with averaging time of 1 s) when the ORD wavemeter resolution was 0.3 pm measured at *λ* = 1028 m. However, other sources of noise exist, arising for instance, from PBS extinction ratios, SLM mode conversion efficiency, etc. Instead of independently characterizing each noise source, we provide an overall systems noise with the measurement-uncertainty band in Fig. 2c deduced from the statistics of repeated measurements.

## Supplementary information

Supplementary Information

## Data Availability

The data that support these findings are available from the corresponding author upon request.
