# Supplementary material for: High resolution spectral metrology leveraging topologically enhanced optical activity in fibers
Source: Nat Commun. 2020 Oct 16;11:5257. doi: 10.1038/s41467-020-18931-6 (PMC7568529; doi:10.1038/s41467-020-18931-6)
Supplement: Supplementary file 1 — Supplementary Information [file 41467_2020_18931_MOESM1_ESM.pdf]

High Resolution Spectral Metrology Leveraging Topologically Enhanced Optical Activity in Fibers

Greenberg *et al*.

Supplementary Information

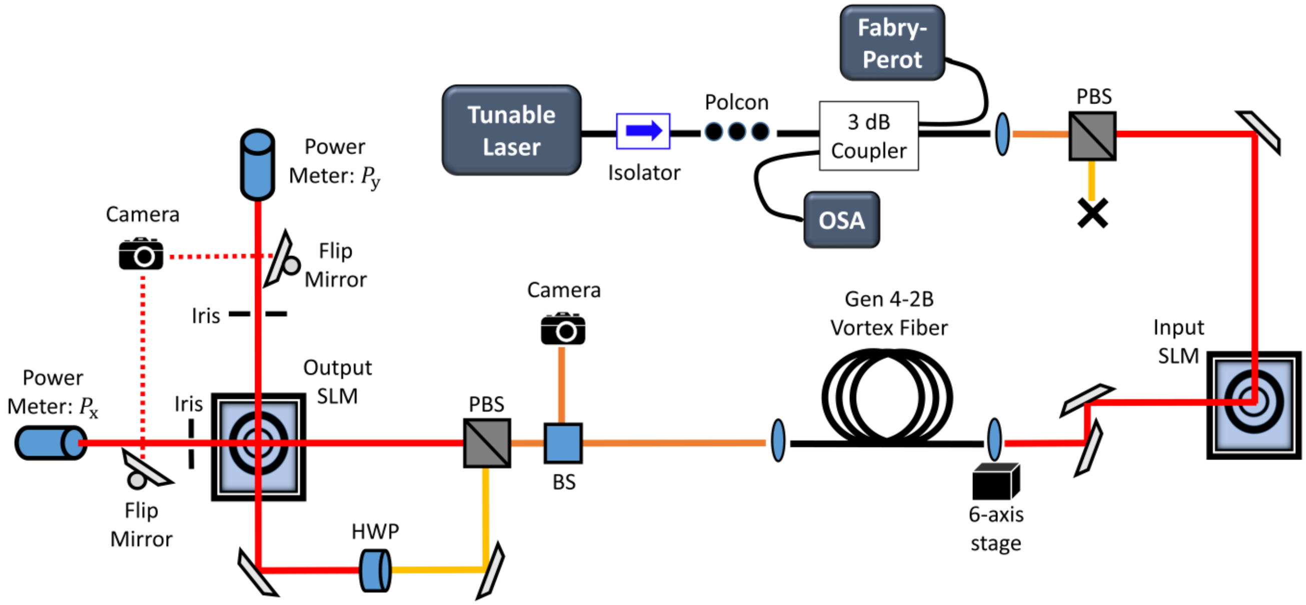

Supplementary Figure 1 | Full ORD Wavemeter setup. Detailed ORD wavemeter (ORDW) setup, used to measure resolving power, expanding on the simplified version portrayed in Fig. 2(a). Free-space light polarization is indicated with color: Red is $\hat{\mathbf{x}}$-polarization, yellow is $\hat{\mathbf{y}}$-polarization, and orange is a combination of $\hat{\mathbf{x}}$- and $\hat{\mathbf{y}}$-polarization. Dashed lines indicate diverted paths using flip mirrors. OSA – optical spectral analyzer; PBS – polarization beam splitter; Polcon – polarization controller; SLM – spatial light modulator; BS – beam splitter; HWP – half wave plate.

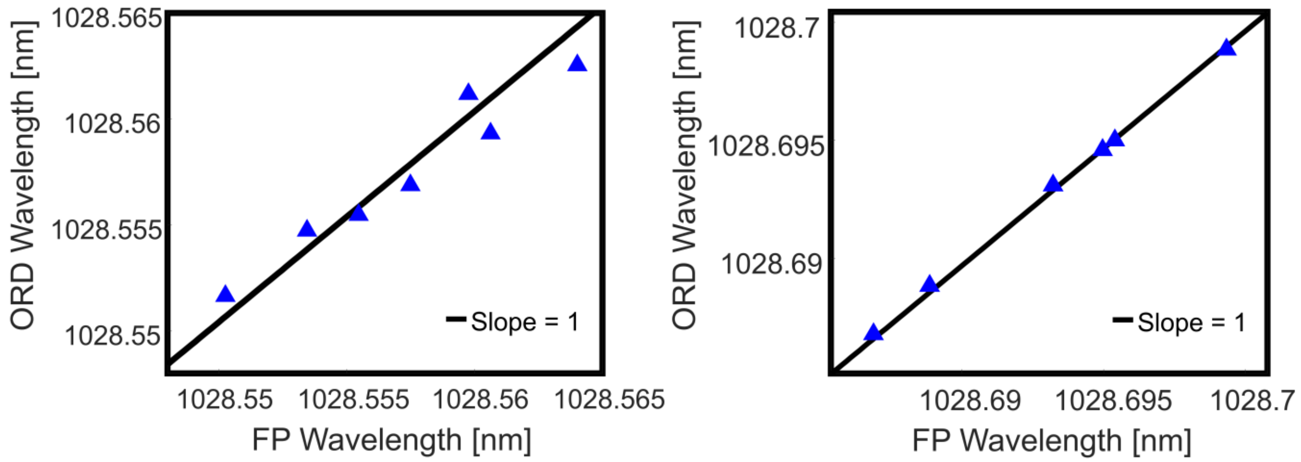

Supplementary Figure 2 | Wavemeter accuracy. Two mode-hop-free spectral regimes used to calculate accuracy of our ORDW spectroscopic γ to λ mapping. Root-squared-mean error (RMSE) is used to determine the deviation of our ORD wavemeter measurements from the Fabry-Perot (FP) ground truth slope=1 line.

Supplementary Note 1: Vortex Fiber Index Profile

Cylindrically symmetric and isotropic optical fibers utilizing a ring-core design fall under a particular classification, known as vortex fibers. Specifically, these fibers are specially designed to mirror the field profile of an OAM beam, which has been found to maximize the effective index splitting (Δn_{eff}) between modes of a given $|\mathcal{L}|$ but opposite circular polarizations, $\hat{\boldsymbol{\sigma}}^{\pm}$ (i.e. SOa and SOaa)[1] . We illustrate an example of such a profile in **Supplementary Figure 3**, which has been measured using an interferometry-based fiber profiler (Interfiber Analysis IFA-100) at 633nm. The fibers used in this work possess a center air-filled hole at the inner boundary of the ring-core. This Δn_{eff} split is due to spin-orbit coupling and the inhomogeneity of the ring-core, which, as predicted by perturbative theory, introduces polarization-dependent perturbations based on $\hat{\boldsymbol{\sigma}}^{\pm}$ and is enhanced by OAM[2,3]. As such, a 4-dimensional Hilbert space of OAM / SAM states exist in vortex fiber [see **Fig. 1(a)**] comprised of degenerate pairs of SOa and SOaa modes, which have effective index differences of $\Delta n_{\text{eff}} > 10^{-4}$ (circular birefringence[4]). A combination of this spin-orbit interaction Δn_{eff} splitting and conservation of angular momentum allows vortex fibers to avoid both non-degenerate and degenerate coupling effects[5,6] between all the modes in their Hilbert space, and have been well known to stably propagate OAM / SAM singlet states[7], as described in Eq. 1.

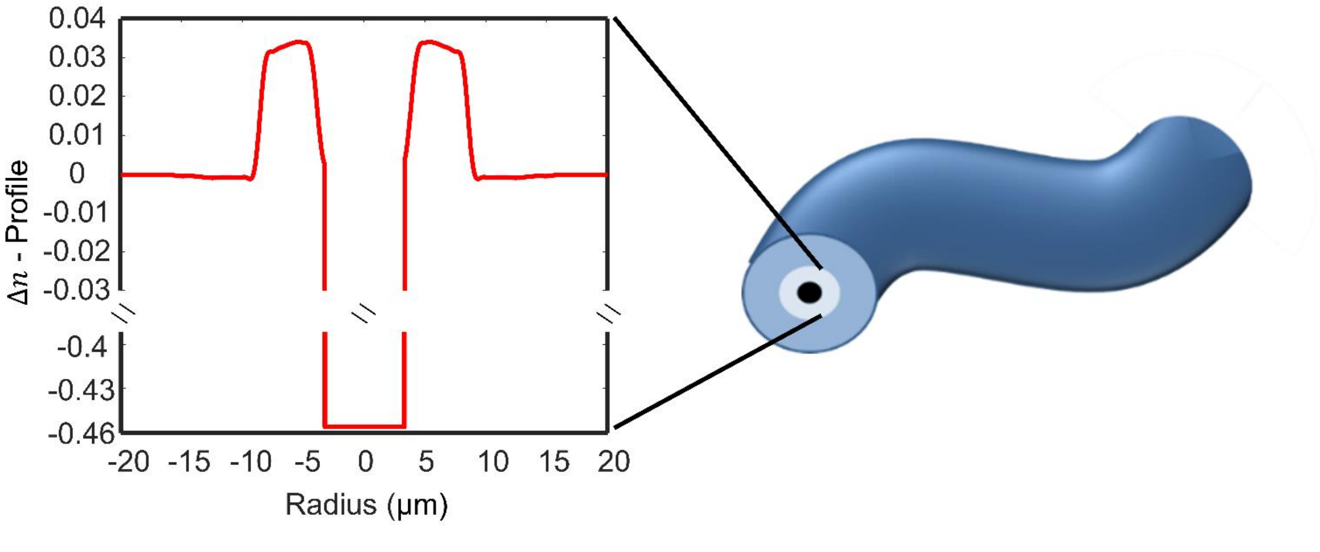

Supplementary Figure 3 | Vortex fiber profile. The vortex fiber (reproduced from supp. ref. [5], 2015, Optical Society of America) refractive index profile (red trace) is designed in the shape of an annular ring, mirroring the intensity profile of an OAM beam. Since the vortex fiber is cylindrically symmetric, the profile can be measured radially in 1-dimension. The Δn refers to the profile's refractive index difference with respect to standard silica.

Supplementary Note 2: Wavelength to Polarization Mapping

The basis of the ORD Wavemeter (ORDW) is the ability to map changes in wavelength directly to a rotation in polarization. The basic optical activity (OA) relation, for a single wavelength, is described in Eq. 4, and explicitly reveals a wavelength-dependence. However, to track how our OA polarization state changes with wavelength from a tunable laser source, we must also account for that fact that our effective index splitting (Δn_{eff}) is wavelength dependent. As such, we take a simple first order Taylor expansion of $\gamma(\lambda)$ around λ_1:

$$\gamma(\lambda) = \frac{2\pi\Delta n_{\mathrm{eff}}}{\lambda} \rightarrow \Delta\gamma = \gamma(\lambda)|_{\lambda_1} - \gamma(\lambda_1) = \frac{2\pi\Delta n_{\mathrm{eff}}}{\lambda_1} + \frac{1}{1!}\frac{d}{d\lambda}\left(\frac{2\pi\Delta n_{\mathrm{eff}}}{\lambda}\right)(\lambda - \lambda_1) - \frac{2\pi\Delta n_{\mathrm{eff}}}{\lambda_1}$$

$$= \frac{d}{d\lambda}\left(\frac{2\pi\Delta n_{\mathrm{eff}}}{\lambda}\right)\Delta\lambda = 2\pi\Delta\lambda\left(\frac{1}{\lambda}\frac{d\Delta n_{\mathrm{eff}}}{d\lambda} - \frac{\Delta n_{\mathrm{eff}}}{\lambda^2}\right) = -\frac{2\pi\Delta\lambda}{\lambda^2}\left(\Delta n_{\mathrm{eff}} - \lambda\frac{d\Delta n_{\mathrm{eff}}}{d\lambda}\right)$$

$$= -\frac{2\pi\Delta n_{\mathrm{g}}}{\lambda^2}\Delta\lambda = \alpha\Delta\lambda \quad (1)$$

We see that from the first order Taylor expansion of our OA, Supplementary Equation 1, we obtain the linear ORD mapping between $\Delta\gamma$ and $\Delta\lambda$ from Eq. 5.

Supplementary Note 3: Modal Purity Analysis and Output Mode Conversion

The ORDW performance relies on our ability to propagate pure OAM states in vortex fiber[5]. To determine modal purity, the output OAM beam's intensity profile is imaged on a Thorlabs DCC1545M camera [see **Supplementary Figure 1**] and interferometric analysis is used to detect interference effects between vortex fiber modes[8]. One way to reduce modal cross talk is with precise input coupling of the beam to the vortex fiber. For this reason, a 6-axis stage (Thorlabs, MAX607L) and "walk-the-beam" method are used for optical alignment [see **Supplementary Figure 1**]. With proper alignment we achieved >20-dB mode purity between OAM modes of $\Delta|\mathcal{L}| \geq 1$. However, our analysis did reveal degenerate coupling caused by fiber perturbations. This degenerate coupling transitioned OAM states ($+\mathcal{L}$, $\hat{\boldsymbol{\sigma}}^{\pm}$) to ($-\mathcal{L}$, $\hat{\boldsymbol{\sigma}}^{\mp}$), resulting in a ~10-dB polarization extinction ratio (PER) for the output beam. Assuming equal fractional power, ϵP_0, degenerately couples into $-\mathcal{L}$-OAM SOa and SOaa states [see **Supplementary Figure 4(a)**], then another linearly polarized superposition state is generated which experiences optical activity in vortex fiber. This degenerate optical activity (DOA) has identical ORD

birefringent strength, since polarization rotates at the same rate [see Eq. 5], but with opposite handedness as compared to the original OA state [see **Supplementary Figure 4(b)**]. The counter-rotation of the OA and DOA alters the projection of power of the output beam into $(\hat{\mathbf{x}}, \hat{\mathbf{y}})$ polarization bins, changing γ [see Eq. 6], and violating the mapping of the ORDW. To rectify this issue, we placed an SLM with an effective $-\mathcal{L}$ fork-pattern at our output [see **Supplementary Figure 1**] to perform a mode conversion technique that eliminates DOA contributions. This technique works by inducing a $-\mathcal{L}$ topological charge on our output OAM beam, transforming the spatial distributions of its OA and DOA components. The OA state's $\mathcal{L}$ topological charge is negated and it is converted into a $\mathcal{L} = 0$ Bessel beam in the far field[9]. Meanwhile, the DOA state's $-\mathcal{L}$ topological charge is doubled, converting it into a $-2\mathcal{L}$ "halo" with a large optical vortex. Adding iris apertures, we block the $-2\mathcal{L}$ halos surrounding the Bessel beams, allowing only light from the OA states to pass through to our photodetectors [see **Supplementary Figure 4(c)**]. Flip mirrors and a camera [see **Supplementary Figure 1**] were used to check that we properly eliminated these DOA halos. In principle, this mode conversion technique is not just limited to filtering out degenerate modes, but could filter out any undesired mode contributions as well (including non-degenerate OAM modes with different and unwanted topological charges). Using mode conversion, we achieved >16-dB PER and eliminated the effects of degenerate coupling on the ORDW performance. We note that several ring-core OAM fibers naturally provide >>16-dB PER, and hence this additional mode filtering is not strictly necessary for our technique to work. Rather, it serves to improve the ORD wavemeter's sensitivity.

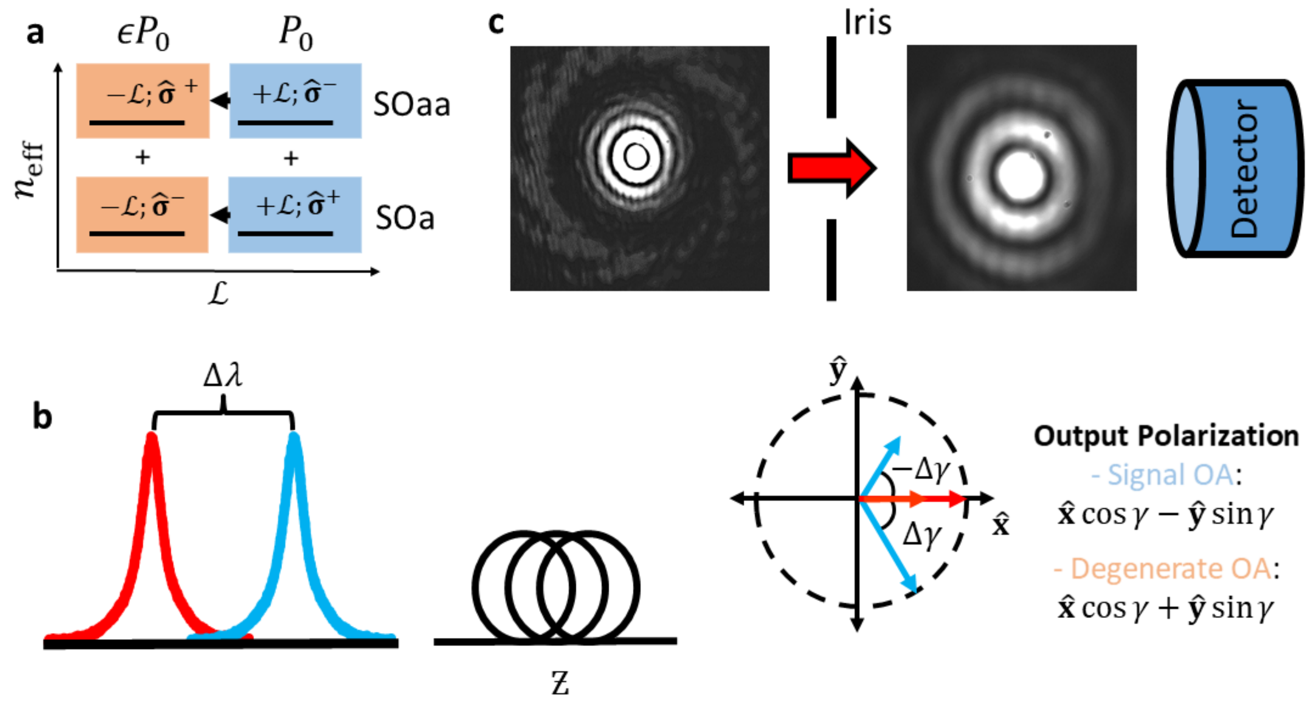

Supplementary Figure 4 | Degenerate coupling effects. (a) Degenerate coupling fills the 4-D vortex fiber mode Hilbert space. The superposition of the highlighted orange states generate a second linearly polarized OAM mode, which experiences degenerate optical activity (DOA) in vortex fiber **(b)** The optical rotary dispersion properties of OA and DOA. For a change in wavelength ($\Delta\lambda$), the OA polarization rotates $\Delta\gamma$, whereas the DOA polarization counter-rotates $-\Delta\gamma$. **(c)** A mode converted beam consisting of an OA Bessel beam and DOA $-2\mathcal{L}$ halo. An iris aperture blocks the $-2\mathcal{L}$ halo, ensuring that only light from the Bessel beam, and thus our original OA state, is measured by the photodetector.

Supplementary Note 4: Spectral Bandwidth Measurements and Simulations

The ORDW setup for measuring spectral bandwidth is the same as described in the Methods section, with a few alterations[10]. In order to control the linewidth of our laser, we add an electro-optic phase modulator (iXblue MPX-LN-10) controlled by an arbitrary waveform generator (AWG, Tektronix AWG7082C). **Supplementary Figure 5** depicts a simplified setup, similar to **Fig. 2(a)**, to illustrate where these additional components are inserted. The spectral bandwidth experiments also requires our PBS to be biased at an angle θ such that power is maximized (P_{max}) in one polarization bin, while minimized (P_{min}) in the other. In our case, we use an alternative method and left $\theta = 0^\circ$, aligned with $(\hat{\mathbf{x}}, \hat{\mathbf{y}})$ polarizations. Instead, we simply adjusted the operating wavelength until our power condition was satisfied. With either method, pre-processing of our setup to meet the visibility power condition is necessary, but could be accounted for during a calibration of the device.

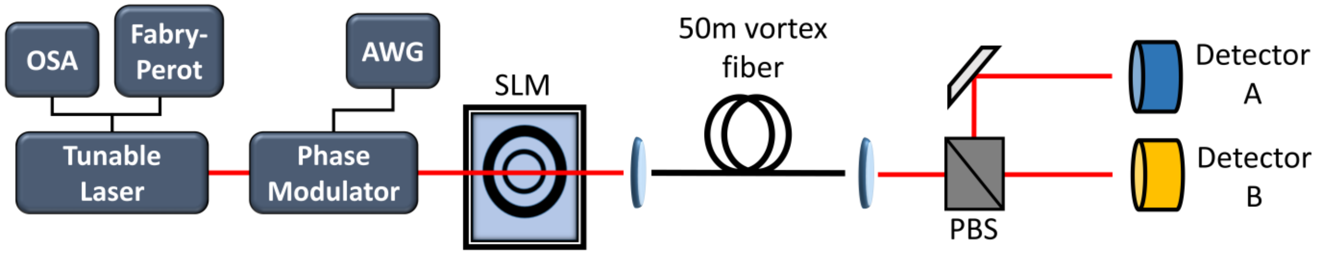

Supplementary Figure 5 | Modified visibility setup. Simplified depiction of the spectral bandwidth experiment with the ORDW. It is identical to the resolving power experiment except that fiber length has been changed to 50 m, and an arbitrary waveform generator (AWG) and electro-optic phase modulator have been added to control the broadening of our spectral lineshape.

If we know the functional form of the ECL, then we can numerically model how visibility reduces with spectral bandwidth FWHM $(\delta\lambda)$. Although the unbroadened ECL spectrum starts with a Lorentzian functional form, this is not maintained when the beam experiences spectral broadening. This is due to the discrete nature of the AWG, which is resolution-limited (125 ps) in controlling phase modulation that generates an ideal broadened Lorentzian (log-Cauchy distribution). As such, the lineshape appears to broaden into a Lorentzian-Gaussian hybrid [**Supplementary Figure 6(a)**], with the function's peak fitting with a Lorentzian but tails trending towards Gaussian behavior. For sufficiently broadened spectra we can assume the functional form of our ECL is actually Gaussian, and therefore we can calculate $P_{\max}$ (in the $\hat{\mathbf{x}}$-bin) to be:

$$P_{\max} = P_0 \int_{\lambda} e^{-(\lambda-\lambda_0)^2/(\delta\lambda^2/4\ln(2))} \cos^2\left[\text{Ƶ}\pi\Delta n_{\mathrm{g}}\left(\frac{1}{\lambda}-\frac{1}{\lambda_0}\right)\right] d\lambda \quad (2)$$

where λ_0 is the operating center wavelength and P_0 is an arbitrary power. Likewise, $P_{\min}$ (in the $\hat{\mathbf{y}}$-bin) can be calculated with Supplementary Equation 2, except with a $\sin^2$. **Supplementary Figure 6(b)** shows how for bandwidths > 0.7 GHz our visibility results converge to a Gaussian numeric model (with 13.7 dB noise), but < 0.7 GHz our visibility deviates and matches closer to a Lorentzian numeric model (with 17.5 dB noise). Note that the data presented in **Supplementary Figure 6(b)** is different than the

data presented in **Fig. 2(d)**. Noise is included to realistically model our photodetector measurements, as $P_{\min} \approx 0$ for a narrowband linewidth would be clearly unphysical.

Figure 2(d) and **Supplementary Figure 6(b)** both show broadening results up to ~2 GHz (~7 pm), which results in, at most, a drop of ~15% visibility. Theoretically, the ORDW can measure spectral bandwidth up until $V \approx 0$, indicating there is a limit to the total spectral broadening it can detect. However, we can overcome this limit and extend the total bandwidth detection. By shortening fiber length and/or using lower order modes the power distribution in Supplementary Equation 2 changes, encompassing a larger span of wavelengths. This would allow us to measure larger changes in $\delta\lambda$ for similar drops in visibility, hence extending the range over which we can broaden our spectrum.

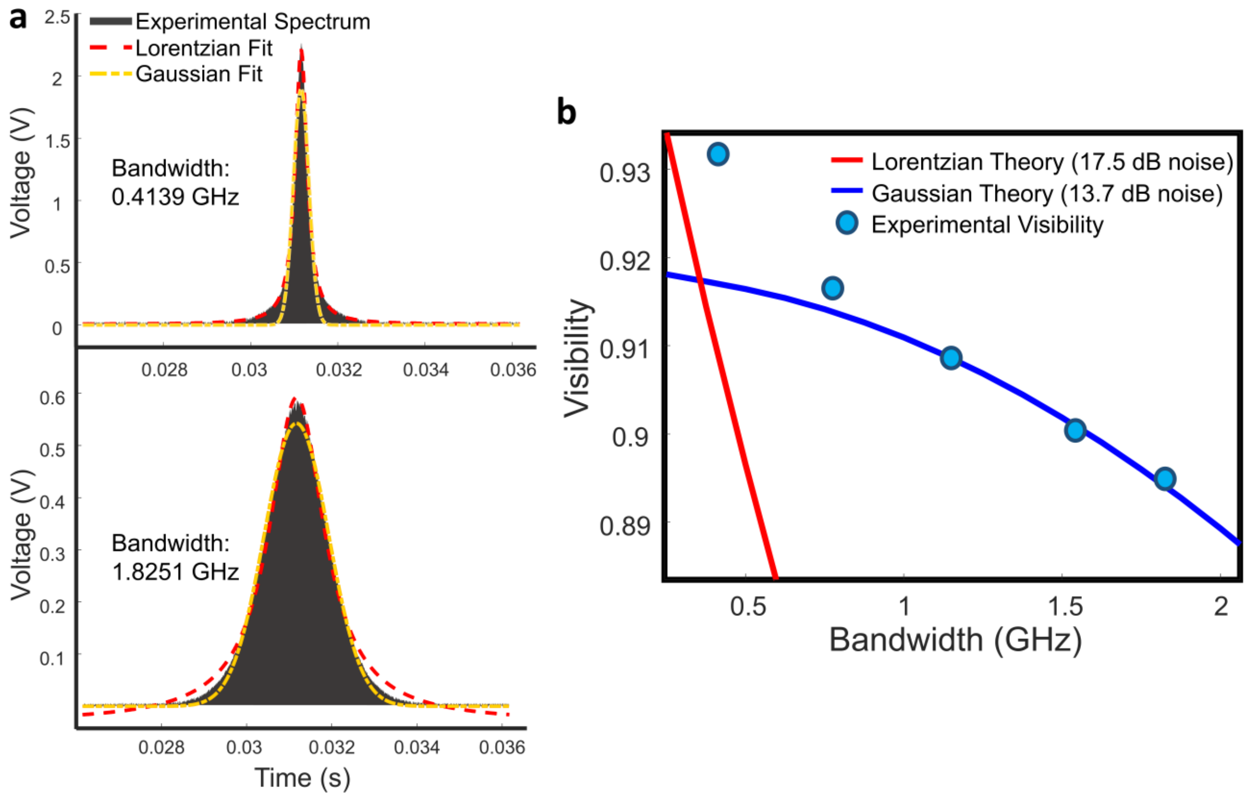

Supplementary Figure 6 | Spectral bandwidth simulations. (a) Lorentzian (red) and Gaussian (yellow) fits applied to both an unbroadened (bandwidth < 0.7 GHz) and broadened (bandwidth > 0.7 GHz) spectrum, measured with a FP. In the unbroadened case, a Lorentzian functional form fits the experimental spectrum better. In the broadened case, a Gaussian functional form fits the experimental spectrum better, especially around the tails. **(b)** Visibility simulations using both Lorentzian and Gaussian numeric models, considering different noise parameters. When the spectrum bandwidth is < 0.7 GHz, the experimental visibility displays Lorentzian behavior, but as the spectrum is broadened, tends towards Gaussian behavior, albeit with a higher noise floor.

Supplementary Note 5: Mechanical and Thermal Perturbations

We determine stability of our ORD mapping by assuring that for a single wavelength (λ_0) its corresponding polarization angle γ_0 does not drift over time. From **Figure 3(a)** we see that the ORDW is far more stable than the PM-fiber (which was spooled and not subjected to external perturbations)[11]. We consider two cases, mechanical and thermal perturbations, where we used a layout identical to that presented in the Methods section, except that we laid out 40 m of vortex fiber on a stirring hotplate (Thermolyne Cimarec 2) and two Ge Thorlabs S122C photodetectors were used to sample power (hence γ). For the mechanical perturbations, we laid out the fiber on a cardboard base, and used the maximum stirring function to vibrate the fiber. Over the course of 5 minutes we measured the OA angle [see **Fig. 3(a)**] and determined that the ORDW remained stable. Next, we introduced thermal perturbations to the vortex fiber, now placed on an aluminum base, with the Cimarec hotplate functionality. For this stability test, we slowly increased temperature of the base from 22 °C to 60 °C, heating the fiber, and measured OA angle as a function of temperature [see **Fig. 3(b)**]. We measured temperature change in real-time using a thermistor connected to a Keysight 34461A Digital Multimeter. We see that OA angle does changes as a clean periodic function with respect to temperature (i.e. time). This predicable and systematic behavior indicates that with careful calibrations, this thermic effect could be accounted for and mitigated.

Supplementary Note 6: SOI Δn_{g} modal dependence

The spin-orbit interaction splitting of effective index between SOa and SOaa vortex fiber modes, Δn_{eff}, has an OAM modal dependence proportional to $\mathcal{L}^2$ in these vortex fibers[12] [see Eq. 2], as determined by perturbative theory[2]. For non-dispersive media we can also approximate that the difference in group index between these modes, Δn_{g}, should have the same dependence. We can numerically calculate Δn_{g} using a finite-difference waveguide solver on the vortex fiber index profile[13]. However, these simulations may be prone to measurement uncertainties, and for this reason we require experimental verification. Since Δn_{g} is a key factor in ORD strength [see Eq. 5], we can calculate it directly from our α calibration factor. By preforming separate calibrations [as discussed in the Methods section] for multiple $\mathcal{L}$-OAM modes, we can compare how Δn_{g} changes with topological

charge. **Supplementary Figure 7** shows results for OAM modes $\mathcal{L} = 10$, 11, and 12, demonstrating excellent agreement of experiment with theory, as the derived Δn_g values from ORD calibration is clearly proportional to $\mathcal{L}^2$.

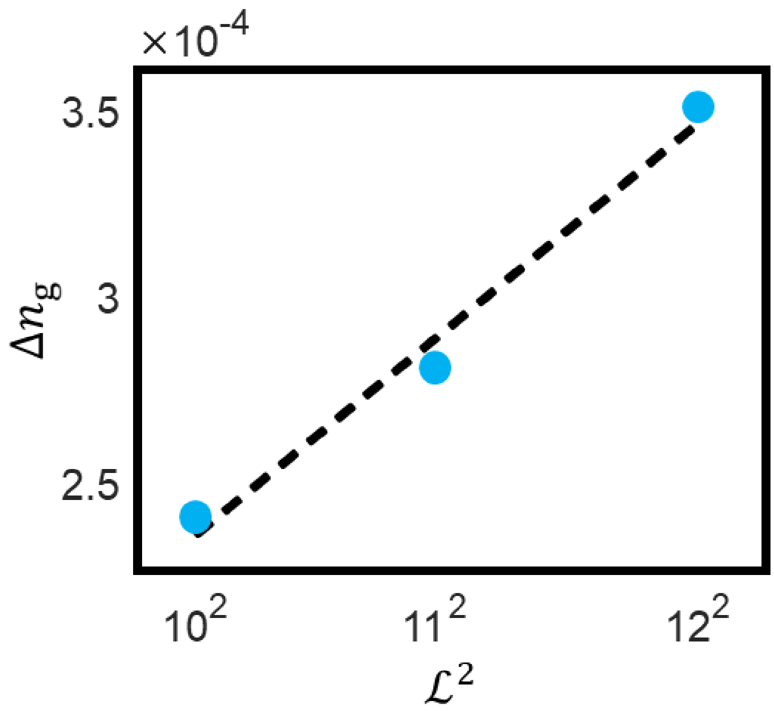

Supplementary Figure 7 | Group index dependence. Experimental validation that the group index difference between SOa and SOaa modes Δn_g in vortex fiber follows the $\mathcal{L}^2$ mode dependence as theoretically predicted by SOI index splitting.

Supplementary Note 7: Figure 4(a) Abbreviations

MEMS - Microelectromechanical systems

OFC – Optical frequency comb

FTS – Fourier transform spectroscopy

BOSA – Brillouin optical spectral analysis

PCA – Principal component analysis

MMF – Multimode Fiber

TFMMI – Tapered fibre multimode interference

DCS – Dual Comb Spectroscopy

Supplementary Note 8: Interference of Gaussian and OAM

In **Figure 4(b)** we illustratively depict the angular momentum through the use of a spiral interference between an OAM and Gaussian beam. The spiral arms, or parastiches, reveal the number of 2π phase rotations comprising the OAM beam's helical phase front, equal to its topological charge $\mathcal{L}$. These spiral patterns were created by reflecting a diagonally polarized beam off a SLM. The beam's $\hat{\mathbf{x}}$-polarized projection is converted to an OAM beam while the $\hat{\mathbf{y}}$-polarized projection remains a Gaussian beam. Passing these beams through a 45° linear polarizer recombines them to generate the spiral interference pattern. The spirals were then imaged on a camera to capture its intensity profile [see **Supplementary Figure 8**].

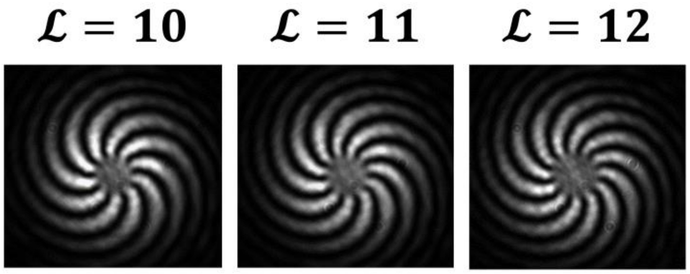

Supplementary Figure 8 | Spiral images. The intensity profiles of spirals created by interfering $\mathcal{L}$ = 10, 11, and 12 OAM beams with a Gaussian beam. The spirals highlight the angular momentum carried by the helical phase front of OAM beams.

Supplementary References:

[1] Ramachandran, S., Gregg, P., Kristensen, P., and Golowich, S.E., "On the scalability of ring fiber designs for OAM multiplexing," *Opt. Express* **23**, 3721-3730 (2015).
[2] Vitullo, D.L.P., Leary, C.C., Gregg, P., Smith, R., Reddy, D.V., Ramachandran, S., and Raymer, M., "Observation of Interaction of Spin and Intrinsic Orbital Angular Momentum of Light" *Phys. Rev. Lett.* **118**, 083601 (2017).
[3] Ramachandran, S., Kristensen, P., and Yan, M., "Generation and propagation of radially polarized beams in optical fibers," *Opt. Lett.* **34**, 2525-2527 (2009).
[4] Greenberg, A.P., Prabhakar, G., and Ramachandran, S., "Single-shot, sub-picometer-resolution wavemeter using topologically enhanced optical activity of OAM fiber modes," in *Conference on Lasers and Electro-Optics*, OSA Technical Digest (Optical Society of America, 2019), paper JTh5A.9.
[5] Gregg, P., Kristensen, P., and Ramachandran, S., "Conservation of orbital angular momentum in air-core optical fibers," *Optica* **2**, 267-270 (2015).
[6] Marcuse, D., *Theory of Dielectric Optical Waveguides* (Academic Press, 1974).
[7] Bozinovic, N., Yue, Y., Ren, Y., Tur, M., Kristensen, P., Huang, H., Willner, A.E., and Ramachandran, S., "Terabit-Scale Orbital Angular Momentum Mode Division Multiplexing in Fibers," *Science* **340**, 1545-1548 (2013).
[8] Bozinovic, N., Golowich, S., Kristensen, P., and Ramachandran, S., "Control of orbital angular momentum of light with optical fibers," *Opt. Lett.* **37**, 2451-2453 (2012).
[9] Liang, Y., Yan, S., He, M., Li, M., Cai, Y., Wang, Z., Lei, M., and Yao, B., "Generation of a double-ring perfect optical vortex by the Fourier transform of azimuthally polarized Bessel beams," *Opt. Lett.* **44**, 1504-1507 (2019).
[10] Greenberg, A.P., Prabhakar, G., and Ramachandran, S., "Measuring spectral bandwidth with OAM fiber mode induced optical activity," in *Conference on Lasers and Electro-Optics*, OSA Technical Digest (Optical Society of America, 2020), paper AF1K.6.
[11] Dimmick, T., "Simple and accurate wavemeter implemented with a polarization interferometer," *Appl. Opt.* **36**, 9396-9401 (1997).
[12] Johnson, S., Ma, Z., Padgett, M.J., and Ramachandran, S., "Measurement of the spin-orbit coupling interaction in ring-core optical fibers," *OSA Continuum* **2**, 2975-2982 (2019).
[13] Pedersen, M.E.V., Kristensen, P., Grüner-Nielsen, L., and Rottwitt, K., "Impact of the Scalar Approximation on the Prediction of the Group Velocity Dispersion." *Jour, Lightwave Tech.* **29**, 3129-3134 (2011).
